# Algae-Boosted Chickpea Hummus: Improving Nutrition and Texture with Seaweeds and Microalgae

**DOI:** 10.3390/foods13142178

**Published:** 2024-07-10

**Authors:** José Matheus, Maria João Alegria, Maria Cristiana Nunes, Anabela Raymundo

**Affiliations:** 1 LEAF—Linking Landscape, Environment, Agriculture and Food Research Center, Associated Laboratory TERRA, Instituto Superior de Agronomia, Universidade de Lisboa, Tapada da Ajuda, 1349-017 Lisboa, Portugal; jmatheussilva@isa.ulisboa.pt (J.M.); cristiananunes@isa.ulisboa.pt (M.C.N.); 2 SUMOL+COMPAL, Rua Dr. António João Eusébio, 24, 2790-179 Carnaxide, Portugal; maria.alegria@sumolcompal.pt

**Keywords:** hummus, pulses, seaweed, microalgae, rheology, food development

## Abstract

The global food industry faces a critical challenge in ensuring sustainable practices to meet the demands of a growing population while minimizing environmental impact. At the same time, consumer awareness and the demand for quality products drive innovation and inspire positive changes in the food supply chain. Aiming to create a more sustainable and nutrient-rich alternative, this study is summarized by characterizing the physical and chemical characteristics of algae-enriched chickpea hummus: an innovative approach to popular food products. The algae-enriched hummuses were developed with an incorporation (6% *w*/*w*) of *Gelidium corneum* and *Fucus vesiculosus* seaweeds and *Chlorella vulgaris* (hetero and autotrophic) microalgae to reveal their technological potential and evaluate the nutritional and rheological characteristics relative to a control hummus (without algae). From a nutritional perspective, the main results indicated that hummus enriched with microalgae showed an increase in protein content and an improved mineral profile. This was particularly notable for the seaweed *F. vesiculosus* and the autotrophic microalga *C. vulgaris*, leading to claims of being a “source of” and “rich in” various minerals. Additionally, the antioxidant activity of hummus containing *F. vesiculosus* and *C. vulgaris* increased significantly compared to the control. From a rheological perspective, incorporating algae into the humus strengthened its structure. The microalgae further enhanced the dish’s elasticity and firmness, thus improving this chickpea-based dish´s overall texture and quality.

## 1. Introduction

Hummus originated in the Middle East and Mediterranean regions [1], is a dish prepared from the legume chickpea (*Cicer arietinum*), and is consumed by Arabs and Jews [2] because it is widely spread in that region. However, its popularity extends far beyond those regions. Nowadays, hummus is enjoyed all over the planet due to the internationalization of gastronomy and culinary traditions. It is not hard to find traditional dishes from various cultures that are being spread rapidly around the globe with mega trends in consumption patterns [3]. Hummus stands out as a strong example in this context. That dish is notable for its delicious flavor, especially resulting from the addition of *tahini* (a sesame seed paste made with olive oil) and for having in its formulation a high percentage of pulses, which are the dried edible seeds harvested from the pod of leguminous plants such as peas, beans, lentils, and chickpeas [4]. As a result of its chickpea content, hummus is more than just a source of protein, but also of polyunsaturated fatty acids, dietary fiber, resistant starch, vitamins, and minerals, especially folate and potassium [5]. This chickpea garnish, considered one of the world’s oldest foods [2], is also a very affordable and easy-to-prepare dish and it combines as a side dish on many occasions, aggregating not only palatability but also nutrition and versatility. It fits into a variety of healthy eating patterns, including the Mediterranean dietary pattern [1], which is focused on consuming a diverse and varied diet based on plant-based foods such as whole grains, seeds, fruits, vegetables, legumes, and nuts [6]. It is noteworthy that pulses are an excellent source of plant-based proteins and will be essential when animal-derived proteins fail to meet the requirements of the global population [7], which is projected to be 10 billion inhabitants in 2050 [8]. Alternative food sources are needed to avoid a global famine [9]. So, why not incorporate an ingredient such as algae into hummus? Algae, including seaweed and microalgae, has a high nutritional potential and creates clean-label products. The possibilities of its applications go beyond enhancing the nutritional profile of hummus when incorporated.

Seaweeds contain many bioactive phytochemicals not commonly found in other foods, are rich in bioactive compounds, such as carotenoids, polyphenols, and vitamins A, C, D, and E, and are receiving more and more attention from many Western countries in recent years [10], making it a great choice to be introduced into food. Its unique properties as food throughout history are also related to the evolution of the human brain [11]. In addition, the sustainable seaweed industry is an important part of the future Bioeconomy which provides food products with more efficient resources [12]. In order to find new food and sustainable resources with a smaller or even positive carbon footprint is a need for an appreciation of the seaweed cuisine, combining that with a science-based gastronomical development and exploring its gastronomic potential [13].

On the other hand, the addition of microalgal biomass is also an interesting tool for providing nutritional supplementation with biologically active compounds, besides coloring purposes [14]. Microalgae is a sustainable source that produces natural colorants in food with low allergenicity, toxicity, and carcinogenicity [15]. They are the answer to the growing need for proteins, carbohydrates, and lipids and may represent a viable route to be followed [16]. The diverse chemical composition of the microalgae biomass and the whole spectrum that this raw material addresses is the main reason why there is a global interest in the exploitation of microalgae-based processes and products [17]. Making algae appealing remains challenging despite its nutritional benefits. The algae industry is niche but aims for global growth by integrating algae into high-quality products to broaden its appeal. Above all, consumers look for products that taste good.

In line with these topics, a traditional chickpea hummus and its variations were developed with an incorporation of (6%) *Gelidium corneum* and *Fucus vesiculosus* seaweeds and *Chlorella vulgaris* (hetero and autotrophic) spray-dried microalgae, with the aim of studying the food potential of these preparations. Since the beginning of the product design, some strategies have been adopted to elevate its concept, including alignment with the SDGs (Sustainable Development Goals) [18] and the development of a marketing plan to influence consumer perception and purchasing decisions [19]. Innovation is essential for attractiveness in the market. The main objective of this study is to understand the effects of algae incorporation into hummus, characterize the products, and explore avenues for their introduction into the market, potentially in convenient, ready-to-eat tube packaging. Physicochemical characteristics, including nutritional composition, antioxidant capacity, rheology, texture, and microscopic structure, were analyzed and compared with control hummus without algae.

## 2. Materials and Methods

### 2.1. Materials

The legumes were supplied by SUMOL+COMPAL (Portugal), a prominent brand in Portugal, renowned for its significant market influence through the distribution of fruit beverages as well as cooked and canned legumes [20].

The algae were supplied by partners within the PRR Agenda—Pacto da Bioeconomia Azul—Algae Vertical project.

**Figure 1:**
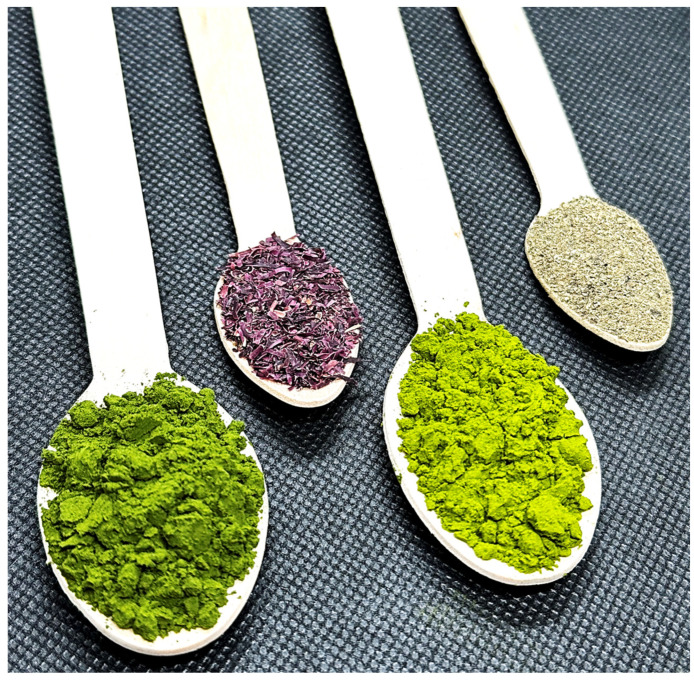
Algae utilized as raw materials in hummus formulations, from left to right: autotrophic *Chlorella vulgaris*, *Gelidium corneum*, heterotrophic *Chlorella vulgaris*, and *Fucus vesiculosus*.

The seaweed *Gelidium corneum* was supplied by Iberagar (Barreiro, Portugal) [21], and the *Fucus vesiculosus* was supplied by Alga+ (Ílhavo, Portugal) [22]. The spray-dried microalgae biomasses of *Chlorella vulgaris* produced under heterotrophic and autotrophic conditions were supplied by Allmicroalgae (Pataias, Portugal) [23]. The algae utilized as raw material is represented in Figure 1 and its nutritional information is in Table 1.

Additional ingredients, including olive oil, sesame seeds, lemon, garlic, balsamic vinegar, and salt, were purchased from local supermarkets in Lisbon, Portugal.

The results of algae incorporation were compared with those of a conventional hummus formulation, also developed within the laboratory, which served as the standard in this research.

### 2.2. Methods

#### 2.2.1. Hummus Preparation

To prepare the hummus, the initial step involved toasting 80 g of white sesame seeds for 5 min at 100 °C to prepare *tahini*. This procedure was carried out in a non-stick frying pan on an induction hob (Taurus, White & Brown, PI400, Barcelona, Spain), with continuous stirring until the seeds turned golden and aromatic and cooled at room temperature. The first step is grinding the ingredients adding only the toasted sesame seeds with 70 g of extra virgin olive oil in a Thermomix food processor (TM6, Vorwerk, Germany) for 2 min at speed 6 until a uniform dark brown paste (known as tahini) was formed.

Subsequently, all remaining ingredients (refer to Table 2) were introduced into the same processor and processed for 5 min at speed 6, resulting in the formation of a thoroughly homogeneous mixture.

For the formulations incorporating algae, four different types of hummuses were prepared in addition to the standard version (without algae), comprising two samples with macroalgae and two with microalgae). The level of incorporations reached 6% (*w*/*w*) in each sample.

The seaweed hummuses were prepared using *Gelidium corneum* or *Fucus vesiculosus*, both of which were previously hydrated in distilled water (1:5 *w*/*v*). The decision to hydrate the seaweed was made purely for sensory reasons in the final product. For the microalgae hummus, *Chlorella vulgaris* (hetero and autotrophic) spray-dried microalgae were used. No pre-preparation, such as hydration, was necessary, as its particle size allows incorporation without sensory or technological obstacles.

To characterize all types of hummuses (control (HC) *Gelidium corneum* (HG); *Fucus vesiculosus* (HF); *Chlorella vulgaris* autotrophic (HA); *Chlorella vulgaris* heterotrophic (HH)), various physicochemical analyses were conducted to understand the impact on the nutritional composition and rheological behavior of the different incorporations.

#### 2.2.2. Hummus Nutritional Composition

Total water content was determined gravimetrically in triplicates using metal melting pots by oven drying, at 100–105 °C (Binder GmbH, ED056, Tuttlingen, Germany), for 24 h until a constant weight was obtained [25,26]. Total ash content was measured by incineration, for approximately 24 h, at 550 °C in a muffle furnace [27].

The determination of crude protein content was made by preparing samples in quadruplicates and using the DUMAS method (Thermo Quest NA 2100 Nitrogen and Protein Analyzer, Interscience, Breda, The Netherlands), which evaluates the nitrogen content, through combustion at 1030 °C [28], using a nitrogen-to-protein conversion factor of 5.7 (indicated to soy bean products).

Crude fat content was evaluated using ether extraction according to the parameters of AOAC 2003.5 [29]. After freeze-drying for 72 h, at −80 °C (ZIRBUS Technology GmbH, VaCo 2, Bad Grund, Germany), a minimum of 1.5 g of each sample was weighed into a 26 mm × 60 mm thimble, and the petroleum ether content was measured by Soxtec extraction (Soxtec System HT 1043/1046 extraction unit–Tecator AB, Höganäs, Sweden). The lipid content was determined gravimetrically in triplicates after 15 min of boiling, 60 min of rinsing, and 15 min of drying, respectively. The total carbohydrate content of hummus was calculated by difference using the values previously analyzed of moisture, ash, protein, and fat.

The quantification of minerals (Na, K, Ca, Mg, P, S, Fe, Cu, Zn, and Mn) was evaluated in triplicates by an Inductively Coupled Plasma Optical Emission Spectrometry (ICP-OES) Thermo Scientific ICAP Series 7000 (Thermo Fisher Scientific, Waltham, MA, USA) [30]. Approximately 0.50 g of sample was weighted into tubes and 12 mL of 37% hydrochloric acid (HCl) and 4 mL of 65% nitric acid (HNO_3_) were added to the tubes. The samples were digested overnight. Deionized water was added to the digested samples to complete the volume of 50 mL. Finally, the samples were transferred to amber glass vials and analyzed in the optical emission spectrometer.

#### 2.2.3. Hummus Bioactivity Assays

To evaluate the in vitro bioactivity of hummus (with and without algae), spectrophotometric methodologies were applied to the analysis of phenolic and antioxidant compounds, using a multi-mode microplate reader CLARIOstar Plus (BMG LABTECH GmbH, Ortenberg, Germany) [31]. Three replicates were performed for each extract in a NUNC 96 microplate.

The extraction consisted of preparing a solution of ethanol and distilled water (80:20 *v*/*v*) and immersing each sample (HC, HG, HF, HA, and HH) in that solution (1:2 *w*/*v*). The mixture was stirred for 120 min at room temperature using a Reax 2 agitator (Heidolph, Schwabach, Germany) with a rotation speed range of 5 rpm. Following that, the samples were centrifuged at 10,000 rpm at 4 °C for 10 min.

After centrifugation, there was the appearance of phase separation. In order to discard the lipid content, it was added 2 mL of hexane into the supernatant of hummus extraction. The liquids were homogenized with a vortex-stirred LBX V05 series (Labbox, Barcelona, Spain) for 1 min + 5 min rest until visually complete separation. After separating the hexane with traces of lipid content, the process was repeated one more time in the same conditions to certify the disappearance of lipids. The final supernatant has been used for the subsequent analysis.

The Folin–Ciocalteu method was performed to quantify the total phenolic compounds (TPC) and were expressed as gallic acid equivalents (GAE) [32,33]. In summary, the extracts (20 µL) were mixed into the microplate with Folin’s reagent (100 µL). After 5 min of reaction, sodium carbonate solution (Na_2_CO_2_ 7%) was added (80 µL). The blend was kept in dark incubation for 2 h at room temperature until the measurement. It was read at 760 nm, using ethanol and distilled water (80:20 *v*/*v*) as blank.

The antioxidant activity of the samples was estimated using two different methods with Trolox equivalents and consequently results in TEAC.

The first was determined through the DPPH (2,2-diphenyl-1-picrylhidrazyl-hydrate) method adapted from Lee et al. (2023) [34], which consists of an organic chemical compound composed of stable free radical molecules capable of identifying the scavenging effect of the samples in the assay [34,35,36]. The ethanolic extractions of the samples (20 µL) were mixed with the methanolic DPPH (180 µL) and kept out of direct light contact for 30 min at room temperature until the measurement in the CLARIOstar Plus reader at 517 nm, adapted from Olufemi (2024) [37], and using ethanol and distilled water (80:20 *v*/*v*) as blank.

The second methodology was performed based on the ferric ion reducing antioxidant power (FRAP) analysis that is efficient in identifying the reduction of ferric chloride (FeCl_3_+) to ferrous chloride (FeCl_2_+). FRAP solution was prepared by mixing acetate buffer, 2,4,6-tripyridyl-s-triazine (TPTZ–10 mM TPTZ in 40 mM HCl), and ferric chloride in a ratio of 10:1:1 inside a sterile plastic tube in that specific order. It was kept in a 37 °C bath for 15 min [38]. The extractions were diluted (1:4 *v*/*v*) in ethanol and distilled water (80:20 *v*/*v*) and added (25 µL) into a NUNC 96 microplate followed by FRAP solution (175 µL). The incubation was in the dark for 30 min at room temperature. The measurement was performed at 595 nm using as blank the same reagent as the DPPH assay.

The obtained values were calibrated against their respective standard curves (1,2,3):

(1) TPC:y=0.016x+0.063,

(2) DPPH:y=25169x+1.2901,

(3) FRAP:y=0.3529x+0.0092.

These curves represent equivalent concentrations of gallic acid for the phenolic compounds assay, and Trolox equivalents for the DPPH and FRAP assays. The curves were constructed using concentrations of 20, 40, 70, 90, 120, and 150 µL/mL of the corresponding reagents. The stock solution of DPPH had an absorbance equivalent to 0.765.

#### 2.2.4. Hummus pH, a_w_, and Color Measurements

The pH monitoring was determined through a digital pH meter, consisting of a potentiometer BASIC 20^PLUS^ (Crison, Alella, Spain) and an electrode (HACH, Rheineck, Switzerland) that was previously calibrated.

Water activity (a_w_) was evaluated at 25 °C in triplicates using the water activity meter (LabMaster-aw Neo, Novasina, Switzerland) that enables measurements under precisely controlled chamber temperature conditions with an electrolytic sensor [39].

Color readings were executed with a colorimeter Chroma Meter CR 400 Series (Konica Minolta Business Technologies, Tokyo, Japan) calibrated to white standards prior to measuring. The data L*, a*, and b* were analyzed in triplicates in a Petri dish with approximately 20 g of each sample. Based on the CIELAB color coordinate system, L* designates brightness (from 0 to 100), a* indicates the values of redness (+60) or greenness (−60), and b* characterizes the values of yellowness (+60) or blueness (−60) [40]. It was also performed the calculation of ΔE, which represents the global level of variation between the control hummus and the hummuses with alga incorporation, being *L*1, *a*1, and *b*1, respectively, the reference values of L*, a*, and b*, and *L*2, *a*2, and *b*2, respectively, the sample values of L*, a*, and b*, depending on the Formula (4):

(4) $$\Delta E=\sqrt{{\left(L1-L2\right)}^{2}+{\left(a1-a2\right)}^{2}+{\left(b1-b2\right)}^{2}}$$

#### 2.2.5. Hummus Rheology Evaluation

Aiming to understand the internal interactions between all the macromolecules of the hummus with seaweed and microalgae compared to the control, linear viscoelasticity analysis was performed using a controlled stress rheometer Haake MARS III (Thermo Fischer Scientific, Waltham, MA, USA), coupled with a UTC Peltier for temperature control.

With the purpose of quantifying the behavior of each different hummus, small amplitude oscillatory shear (SAOS) measurements were conducted using a 20 mm diameter serrated parallel plate–plate sensor system, at 20 °C, with a frequency range of 0.01–100 Hz [41] and a stress of 3 Pa previously determined as linear viscoelastic region through a stress sweep analysis.

The mechanical spectrum, obtained from the frequency sweep tests, was acquired with G′ representing the storage modulus (elastic component) denoting the hummus’ capacity to store and recover energy during deformation. Simultaneously, G″ represented the loss modulus (viscous component), elucidating hummus’ tendency to dissipate energy during the deformation [42]. Both moduli were examined as functions of frequency. G′ at a frequency of 1 Hz was specifically selected for the subsequent analysis of variance.

The decision to conduct the analysis at 20 °C considers that consumers typically purchase these hummus varieties packaged in tubes at room temperature in supermarkets.

#### 2.2.6. Hummus Texture Analysis

Texture Profile Analysis (TPA) in penetration mode was performed in a TA-XT plus texturometer (Stable Micro Systems, Goldaming, UK) with a load cell of 5 kg. The TPA was executed with a Perspex cylindrical probe with 10 mm diameter at 1 mm/s until a 20 mm depth. The test, also known as the “two bite test”, consisted of two consecutive cycles with 5 s between cycles with performance in quintuplicate at 20 °C. The samples were fixed on glass containers with 6 cm diameter and 5.5 cm height, being filled to 4 cm height [43].

From this analysis, the measured parameters were (a) firmness (N), determined by measuring the peak force of the first cycle; (b) adhesiveness (-N.s), calculated by the negative area; (c) cohesiveness refers to the ratio of the area of the second cycle to the area of the first one [44].

#### 2.2.7. Hummus Microscopy Analysis

A microscopic evaluation by scanning electron microscope (SEM, TM3030^PLUS^ TabletUp Microscope-Hitachi, Tokyo, Japan) was performed to observe the structural integration of hummus matrices.

Separately, the samples (HC, HG, HF, HA, and HH) were placed in the specimen holder, and the freezing model was applied at a temperature of −14 °C. The observations were made in five different magnifications (1000×, 500×, 200×, 50×, and 30×) being considered as a better interpretation of the 50× amplification, with a scale bar of 2 mm [45].

#### 2.2.8. Statistical Analysis

GraphPad Prism software (version 5) was used to perform some statistical analysis, including the variance (ANOVA) and the Tukey test to compare three or more samples. Results were considered significantly different when *p*-values were inferior to 0.05.

## 3. Results and Discussion

### 3.1. Nutritional Characterization and Mineral Profile

#### 3.1.1. Nutritional Composition

For the nutritional characterization, analysis was conducted to determine the levels of moisture, ash, protein, fat, and carbohydrates. All values are detailed in Table 3.

Regarding the moisture results, it is noteworthy that the standard hummus (HC), *Gelidium corneum* hummus (HG), and *Fucus vesiculosus* hummus (HF), exhibited higher moisture values. This can likely be attributed to the hydration process carried out before incorporating the algae into the hummus matrix. Furthermore, the polysaccharide chains found in macroalgae exhibit excellent hydrophilicity, potentially forming a network capable of promoting water retention and facilitating the formation of hydrogen bonds among water molecules [46]. HC and HG exhibited no significant differences (*p* > 0.05) in moisture content, with values of 62.94 and 61.31 g/100 g, while HF showed a significant decrease in moisture (55.81 g/100 g) with a *p*-value < 0.05. Both microalgae-containing hummuses (HA and HH) showed similar percentages without significant differences (*p* > 0.05) and, in the general overview of the samples, they exhibited the lowest humidity values (51.77–51.86 g/100 g).

The ash measurements, obtained as a result of the carbonization of the samples, showed no significant differences between all the hummus types, with values ranging from 2.19 to 2.68 g/100 g.

The DUMAS analysis for crude protein revealed higher values for microalgae hummuses and lower values for seaweed hummuses. Specifically, the results were 9.70% for *C. vulgaris* autotrophic hummus, 8.65% for *C. vulgaris* heterotrophic hummus, 7.23% for control hummus, 7.13% for *G. corneum* hummus, and 6.94% for *F. vesiculosus* hummus. These results were expected due to the high protein content of the microalgae *Chlorella*. Protein content was separately measured for each pure algae using DUMAS analysis, resulting in values of approximately 52% for *C. vulgaris* autotrophic and 32% for *C. vulgaris* heterotrophic. These values exceeded those reported by Batista et al. (38% for *C. vulgaris* autotrophic) [14] but were consistent with the technical specifications provided by the company (Table 1). These evaluations justify the higher values and the significant differences (*p* > 0.05) observed for microalgae hummuses compared to the control. *Fucus vesiculosus* hummus, on the other hand, exhibited the lowest protein values, consistent with both the individual analysis by DUMAS (approximately 10%) and the information provided in the Alga+ technical sheet (Table 1).

The fat content demonstrated the highest values for both microalgae hummus types (HA and HH), with no significant differences (*p* > 0.05) between them (13.97 and 13.77 g/100 g, respectively). In contrast, the seaweed hummus types (*G. corneum* and *F. vesiculosus*) presented the lowest values overall, with no significant differences between each other (8.18 and 9.03 g/100 g, respectively). The addition of olive oil (Oo), as expected, resulted in fat-level growth in all formulations. The technical information provided by the suppliers (Table 1) justifies the higher fat values for microalgae hummuses and lower values for seaweeds, especially *F. vesiculosus*.

The carbohydrate analysis suggested the highest value for *F. vesiculosus* hummus (25.92 ± 1.47 g/100 g), with significant differences observed. This may be attributed to the highest percentage of dietary fiber presented in Table 1 (41.5%), as fibers are also carbohydrates and serve as sources of energy. The hummuses with *G. corneum, C. vulgaris* autotrophic, and *C. vulgaris* heterotrophic had median values between each other without significant differences (*p* > 0.05). The standard hummus holds the lowest value of carbohydrate content (17.59 ± 0.12 g/100 g). The availability of *G. corneum* carbohydrates was likely affected by the non-thermal hydration process. Investigations by Cebrián-Lloret et al. [24] showed that a temperature of 130 °C is necessary to release the polysaccharide agar from the tough cell walls of *G. corneum*, as it is insoluble in cold water. Since hummus is not treated at high temperatures, the long-chain carbohydrates in this alga are unlikely to be extracted, preventing these gelling agents from being released into the hummus matrix, and does not demonstrate high values in carbohydrates.

#### 3.1.2. Mineral Profile

The mineral composition of the hummus displayed notable variability, as detailed in Table 4. Specifically, the formulation without algae incorporation serves as a significant “source of” calcium (Ca), phosphorus (P), iron (Fe), copper (Cu), and manganese (Mn), according to Regulation (EC) No 1924/2006 [47].

The addition of algae significantly enhanced the nutritional profile of hummus, complementing the inherent mineral richness already present, most likely due to the nutritional benefits of the chickpea legume, thereby augmenting various nutritional claims associated with formulations incorporating seaweed or microalgae.

Specifically, *Gelidium corneum* incorporation was identified as a source of potassium (K), magnesium (Mg), phosphorus (P), iron (Fe), and copper (Cu); *Fucus vesiculosus* incorporation as a source of potassium (K), calcium (Ca), magnesium (Mg), and phosphorus (P); Autotrophic *Chlorella vulgaris* incorporation contributed as a source of calcium (Ca), magnesium (Mg), and zinc (Zn); heterotrophic *C. vulgaris* incorporation served as a source of potassium (K), magnesium (Mg), phosphorus (P), iron (Fe), copper (Cu), and manganese (Mn).

It is noteworthy to highlight the presence of specific mineral quantities within the hummus formulations. Zinc (Zn), an essential mineral crucial for numerous metabolic pathways, such as carbohydrate synthesis and protection against oxidative stress [48], exhibits a significant prominence (*p* < 0.05) solely in the autotrophic microalgae incorporation (2.66 ± 0.08 mg/100 g).

Conversely, potassium (K), vital for processes like protein synthesis, nerve impulses, and maintenance of intercellular fluid balance [48], does not emerge as a “source of” potassium in autotrophic *C. vulgaris* incorporation, despite being identified as such in all other algae incorporations.

Overall, the addition of *F. vesiculosus* seaweed and autotrophic microalgae *C. vulgaris* enables claims of being “rich in” specific minerals.

*F. vesiculosus* incorporation was noted for its richness in iron (Fe), copper (Cu), and manganese (Mn). The high values of iron and manganese of *F. vesiculosus* are in accordance with the characterization carried out by Nova et al. (2023) [49], which analyzed and compared the chemical composition and the antioxidant potential of five algae cultivated in fully controlled closed systems. With regard to these iron levels, the consumption of this brown algae, if not in excess, could serve as a potential strategy to address mineral deficiencies in certain diets. Iron is essential for various biological functions, including energy metabolism. Iron deficiency is a widespread issue globally, often leading to serious anemia [50].

Similar significance can be attributed to manganese, as it plays a crucial role in neurotransmitter production and the regulation of reproductive hormones [51], for instance.

From the same perspective, autotrophic *C. vulgaris* incorporation exhibited richness in phosphorus (P), iron (Fe), copper (Cu), and manganese (Mn). These high values for specific minerals are in accordance with studies of Ylmaz-Ersan and Suna (2024) [52], which compared the nutritional profile of probiotic cheeses enriched with different microalgae. Considering that the incorporation of *C. vulgaris* microalgae has been associated with claims of being a calcium source and rich in phosphorus, it is crucial to emphasize the connection of these minerals with the body’s regulatory mechanisms [53], particularly in their role in maintaining bone and dental health [54].

### 3.2. Phenolic Compounds and Antioxidant Capacity

**Figure 2:**
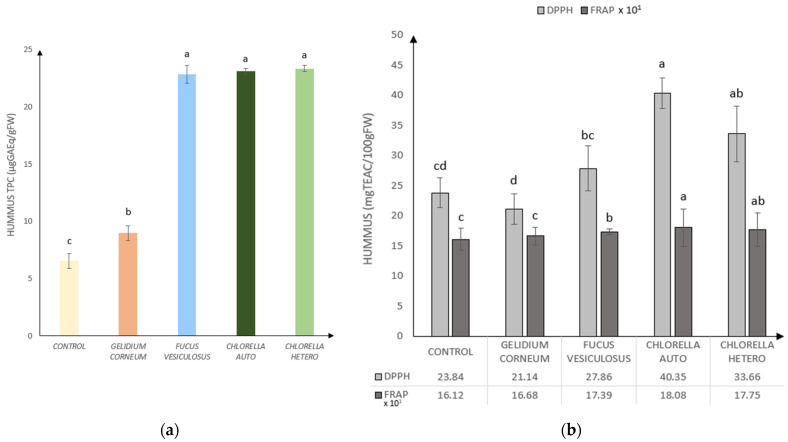
Bioactivity of control hummus and formulations with *Gelidium corneum*, *Fucus vesiculosus*, and *Chlorella vulgaris* (heterotrophic and autotrophic) (6% *w*/*w*): (**a**) Hummus’ Total Phenolic Compounds (TPC) in µgGAEq/gFW; (**b**) Hummus’ Antioxidant Analysis by DPPH and FRAP × 10^1^ in mgTEAC/100 gFW. Error bars indicate the standard deviations from the repetitions. Different letters correspond to significant differences (*p* < 0.05).

To measure the total phenolic composition and antioxidant capacity of each hummus type, analyses of Total Phenolic Content (TPC), Ferric Reducing Antioxidant Power (FRAP), and 2,2-Diphenyl-1-picrylhydrazyl (DPPH) were carried out, according to results in Figure 2a,b.

The Total Phenolic Content (TPC) analysis, as depicted in Figure 2a, indicated by the lowest value for the standard (Control), with subsequent increases observed for all algae incorporations. Specifically, there was a notable enhancement of 137% in *G. corneum*, 348% in *F. vesiculosus*, 353% in autotrophic *C. vulgaris*, and 357% in heterotrophic *C. vulgaris* formulations.

Consistent with the TPC results, the FRAP and DPPH assays (depicted in Figure 2b) revealed an increasing antioxidant activity associated with microalgae incorporations compared to seaweeds. Notably, *F. vesiculosus* hummus exhibited higher values than *G. corneum*, suggesting it remains a better choice alongside microalgae in terms of antioxidant potential.

### 3.3. pH, a_w_, and Color Interpretations

The pH values ranged from 4.54 in standard hummus to 4.82 in autotrophic *C. vulgaris* hummus, with an assumption of similar shelf-life behavior across all applications. Recorded values are outlined as follows: (HC) 4.54; (HG) 4.76; (HF) 4.74; (HA) 4.82; (HH) 4.72. Regarding water activity (a_w_), it was noted that the incorporation of algae did not significantly impact water activity (*p* > 0.05). The recorded values were as follows: (HC) 0.977; (HG) 0.982; (HF) 0.978; (HA) 0.975; (HH) 0.973.

Color measurements resulted in determinations of L*, a*, and b*, along with values of total color difference, ΔE, as presented in Table 5.

**Figure 3:**
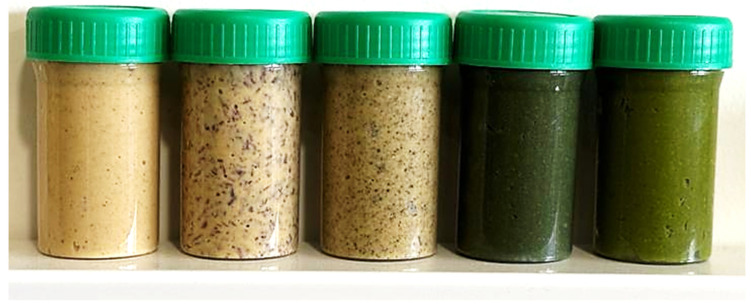
Hummus formulations from left to right: control (HC), *G. corneum* (HG), *F. vesiculosus* (HF), *C. vulgaris* (HA), and *C. vulgaris* (HH).

The results revealed significant differences in the brightness component (L*), with only the standard and *Fucus* hummus showing no significant differences (*p* > 0.05). Regarding a* parameter, which represents the green (negative) to red (positive) colors, positive results were noted for the control and *G. corneum* hummus, indicating a tendency towards redness. In contrast, *F. vesiculosus* hummus exhibited values close to zero. Microalgae-based hummus, on the other hand, displayed negative results, corresponding to greenness. These results represented a closer approximation to red color for seaweed incorporation and a tendency towards green color for microalgae incorporation. The b* parameter, representing the blue (negative) to yellow (positive) colors, exhibited significant differences between all samples (*p* < 0.05). Higher values were observed for the control, *F. vesiculosus*, and heterotrophic *C. vulgaris* (closer to yellow) while lower values were recorded for *G. corneum* and autotrophic *C. vulgaris* (further from yellow). The results of total color difference ΔE, relative to the control sample, demonstrated the highest values for the autotrophic *C. vulgaris* incorporation (31.11). Following this, heterotrophic *C. vulgaris* incorporation ranked second (17.95), *G. corneum* incorporation placed third (12.85), and *F. vesiculosus* incorporation had the lowest value (5.07). All recorded values are higher than 5, indicating detectable color differences by untrained observers [55]. This suggests that concerning visual color and overall level of variance, samples containing seaweed are more similar to the control than those incorporating microalgae (Figure 3). Among these, the lowest values were observed for *F. vesiculosus*, indicating that the incorporation of this seaweed resulted in a color most resembling the standard.

### 3.4. Rheology Behavior

**Figure 4:**
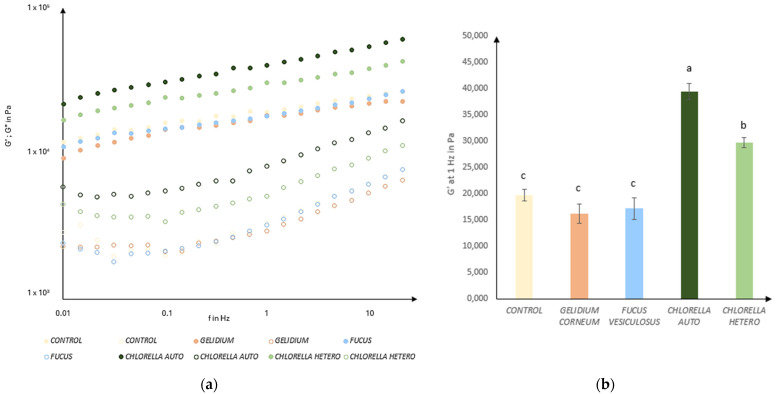
(**a**) Mechanical spectra of control hummus, and with the incorporation of *Gelidium corneum, Fucus vesiculosus,* and *Chlorella vulgaris* (auto and heterotrophic) (6% *w*/*w*). G′ (storage modulus —filled symbol) and G″ (loss modulus—open symbol); (**b**) Values of G′ at 1 Hz for all hummuses. Error bars indicate the standard deviations from the repetitions. Different letters correspond to significant differences (*p* < 0.05).

The rheological evaluation of the hummus revealed similar behavior across all samples through the frequency sweep analysis (Figure 4a), characteristic of a gel-like structure. This indicates that all the samples have the same structural type, with both viscoelastic moduli showing frequency dependence and G′ (elastic modulus) higher than G″ (viscous modulus) for the tested frequency range. Furthermore, samples with microalgae incorporation presented higher values of the viscoelastic functions compared to control and seaweed-containing samples. For a more detailed comparison focusing on the elastic component, G′ values at a frequency of 1 Hz were compared (Figure 4b).

The control, *G. corneum,* and *F. vesiculosus* incorporations had no significant differences between them (*p*-value > 0.05). This leads to the conclusion that hummuses containing seaweed (6% *w*/*w*) exhibit similar viscoelastic properties when compared to a standard spread. This can be attributed to the use of seaweeds with no thermal processing which would release gelling agents through hydration if heated [56].

In the absence of thermal processes, the hydration of the seaweed could have influenced its dispersion within the hummus matrix, thus impacting its rheological properties. Without hydration, seaweed hummus could provide a gritty mouthfeel due to its rigid particles and could lead to consumer rejection [57]. Non-thermal processes for seaweed hydration were selected in order to preserve their colors, flavors, and nutritional quality [58]. This decision was made to ensure better comparison with the properties of microalgae, which also underwent non-thermal processing.

Both microalgae incorporations showed a higher degree of structural reinforcement compared to all other samples, with significant differences observed between them (*p* < 0.05). This can be attributed to the superior protein content of the autotrophic variant compared to the heterotrophic one (Table 1). Specifically, at a frequency of 1 Hz, the gel containing autotrophic *C. vulgaris* displayed the highest level of structuring, expressed by G1_Hz_’ = 3.9 × 10^4^ Pa, followed by the heterotrophic variant (G_1Hz_’ = 3.0 × 10^4^ Pa). These observations align with studies carried out by Batista et al. (2011) [59], which noted that the incorporation of microalgae into a pea protein and starch system led to a tighter integration within the gel network. In both cases, this addition resulted in denser microstructures with improved rheological properties.

As expected, post-preparation, the hummus turned into a homogeneous and thick paste primarily composed of a structure formed through the gelatinization of chickpea starch and the gelation of proteins. This occurs due to a physical competition between these macromolecules for water absorption [60]. Additionally, the importance of the protein’s ability to emulsify is emphasized by the presence of lipids, which are at a level of 27.1 g per 100 g in the control formulation.

The surface activity of proteins is increased after its partial denaturation induced by temperature treatments [61]. Plant-based proteins such as soy, pea, chickpea, and oats are characterized by a predominant globular structure. Upon denaturation, these proteins form a gelled protein network [62]. Similarly, microalgal proteins exhibit the unfolding of their globular structure in response to processing conditions. The aggregation behavior and solubility of microalgal proteins are influenced by factors such as temperature, ionic strength, and pH [63]. In addition to their protein content, microalgae also contain storage carbohydrates (e.g., starch) and structural carbohydrates (e.g., fiber) due to their complex cell wall composition. These components play a significant role in structure reinforcement [64], contributing to the enhanced viscoelasticity of the microalgae-containing hummus.

Microalgae also present favorable surface-active properties, with their proteins displaying a notable resilience to pH changes compared to other hydrocolloids utilized as stabilizers. Consequently, they demonstrated enhanced efficacy in stabilizing oil–water interfaces [65]. This capacity is ascribed to the low isoelectric point and relatively strong resistance to ionic strength found in algal proteins. The increasing comprehension of their emulsification and stabilization capabilities positions microalgal proteins as increasingly sought-after emulsifiers and stabilizers [61]. This attribute could offer a significant advantage in the incorporation of microalgae into hummus formulations.

### 3.5. Texture Results

Through Texture Profile Analysis (TPA), hummuses were characterized by parameters including firmness (N), adhesiveness (-N.s), and cohesiveness.

The incorporation of autotrophic microalgae *C. vulgaris* resulted in higher values of firmness and adhesiveness (Table 6), followed by the heterotrophic variant, with significant differences (*p* < 0.05) observed between all samples. The reinforcement attributed to the addition of microalgae was also noted by Wang et al. (2023) [66], who reported an increase in hardness due to the addition of Spirulina (*Limnospira platensis*) in soy protein hydrogels subjected to high-speed shearing homogenization. In terms of seaweed, both *G. corneum* and *F. vesiculosus* showed significantly lower values compared to microalgae (Table 6). Their values were similar to the standard, with no significant differences observed in firmness (*p* > 0.05). However, adhesiveness is similar between *F. vesiculosus* and the control, while *G. corneum* obtained the lowest value for this parameter. These findings suggest that the incorporation of *G. corneum* in hummus resulted in less tendency for the particles to cling to each other. This behavior is likely due to the absence of heat treatment in any of the formulations. Heat treatment would be necessary to extract the agar gelling agent present in the structure of this alga and similar ones such as *Gracilaria gracilis*. It would require heating at 90–100 °C for 2 h [65,66,67]. From an industrial perspective, this treatment is not viable because the hummus matrix does not typically require high temperatures during preparation. Applying such high temperatures solely for formulations with seaweeds is not justified.

**Figure 5:**
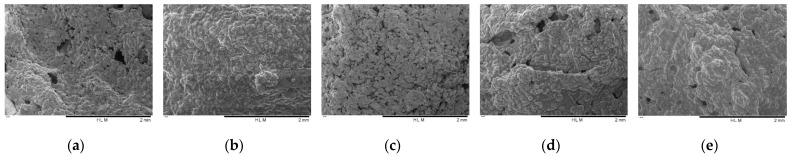
Scanning electron microscope images from hummus formulations (50× amplification, with a scale bar of 2 mm). (**a**) Control Hummus (HC); (**b**) *Gelidium corneum* 6% Hummus (HG); (**c**) *Fucus vesiculosus* 6% Hummus (HF); (**d**) Autotrophic *Chlorella vulgaris* 6% Hummus (HA); and (**e**) Heterotrophic *Chlorella vulgaris* 6% Hummus (HH).

Cohesiveness, which relates to the ability of a sample to recover from deformation, or more specifically, the resistance of the sample to a secondary deformation compared to its reaction to the initial deformation [68], yielded high values (Figure 5) for control, *F. vesiculosus* and heterotrophic *C. vulgaris* hummus (*p*-value > 0.05). The incorporation of autotrophic *C. vulgaris* showed no significant differences in cohesiveness compared to the heterotrophic (*p* > 0.05) and showed a slight decrease compared to the control/seaweed’s hummuses (*p* < 0.05). *G. corneum* hummus presented the lowest values of cohesiveness.

It is important to note that the texture results align with the frequency sweep analysis and may also be related to the higher values of protein (Table 3), suggesting a direct proportional relationship between these two parameters. For instance, the autotrophic *C. vulgaris* presented the highest values for protein content, frequency sweep analysis, firmness, and adhesiveness. In that case, as Bernaerts et al. (2019) [64] elucidated in their review, photoautotrophic microalgae are abundant in structural biopolymers like proteins and storage polysaccharides. These components have the potential to significantly alter the rheological properties of the enriched food product and offer compelling technological functionalities.

On the other hand, seaweeds are known for being excellent structuring components primarily due to their long-chain polysaccharides, rather than their protein content. However, in this research, the utilization of non-thermal treatment prevented the reinforcement of hydrocolloids that could have been extracted from these polysaccharides, thus inhibiting the formation of robust gels [69].

### 3.6. Microscopy

The microscopic evaluation by SEM confirmed the compact microstructure of *C. vulgaris* (autotrophic and heterotrophic) incorporations, as depicted in Figure 5d,e. This observation aligns with expectations, given the high levels of protein content, firmness, adhesiveness, cohesiveness, and viscoelastic properties associated with these formulations.

The hummus containing *F. vesiculosus* (Figure 5c) displayed a structure most similar to the control (Figure 5a), suggesting it could serve as a viable alternative for consumers seeking products enriched with minerals and antioxidant compounds while maintaining similar attributes in terms of firmness, color, and adhesiveness.

The hummus with *G. corneum* (Figure 5b) displayed no discernible differences in terms of microscopy. Despite appearing to possess a more compact structure, the results of the TPA tests (specifically, cohesiveness) indicated that its structure was actually more fragile and prone to deformation. As discussed previously, this fragility and tendency for deformation can be attributed to the absence of heat treatment, which would typically release the chains of agarose, in addition to the hydration of the seaweed. It is also possible that the particle size of *G. corneum* may not have absorbed water as effectively as in *F. vesiculosus* sample.

## 4. Conclusions

Exploring vegetable sources from seaweed, microalgae, and pulses offers a promising solution to food sustainability and nutrition challenges. With a growing global population and increasing environmental concerns, diversifying protein sources beyond traditional animal products is crucial. The addition of seaweed, especially *Fucus vesiculosus*, provides a rich content of essential minerals, such as iron, copper, and manganese, as well as antioxidants and phenolic compounds, which are vital for a balanced diet. Incorporating microalgae, particularly autotrophic *Chlorella vulgaris*, offers a diverse array of minerals including phosphorus, calcium, magnesium, iron, copper, and manganese. Additionally, microalgae supply protein, antioxidants, and phenolic compounds act as natural structuring agents. While heterotrophic *C. vulgaris* may not be as prominent as its autotrophic counterpart, it still possesses notable qualities, particularly in terms of antioxidant activity and mineral content. Conversely, the incorporation of *Gelidium corneum* does not significantly affect any analyzed parameter, aside from its aesthetic characteristics, with the red algae particles in suspension potentially appealing to certain consumers. Overall, the algae used in this study offered sustainable protein sources, introduced new textures and colors, and enhanced the nutritional profile of hummus. This marks a significant advancement in the food industry toward more diverse and eco-friendly options, with their nutritional value being particularly noteworthy as plant-based alternatives. The addition of seaweed or microalgae to hummus alters its physicochemical attributes, although a formal sensory analysis was not performed. Initial evaluations of taste, texture, and consumer appeal were based on informal consortium meetings. Future research will include formal sensory analyses to thoroughly assess how algae affect the properties of hummus.

## Figures and Tables

**Table 1:** Nutritional composition of the algae used in this research. *Gelidium corneum* from Cebrián-Lloret [24]. *Fucus vesiculosus* from product specification sheet (Alga+). *Chlorella vulgaris* auto and heterotrophic from product specification sheet (Allmicroalgae).

Algae	Protein (g/100 g)	Fat (g/100 g)	Carbohydrates (g/100 g)	Dietary Fiber (g/100 g)
*Gelidium corneum*	19.4	4.7	35.2	nd ^1^
*Fucus vesiculosus*	14.7	2.2	10.0	41.5
*Chlorella vulgaris* (heterotrophic)	50.0	9.5	14.2	15.2
*Chlorella vulgaris* (autotrophic)	38.3	14.2	21.8	17.7

^1^ Not determined.

**Table 2:** Standard hummus formulation (without algae).

Ingredients	Amount (in g)
*Tahini*	150 (70 of Oo and 80 of Ss) ^1^
Canned chickpeas	400
Lemon juice	50
Garlic	5
Balsamic vinegar	5
Salt	6
Aquafaba (from the canned chickpeas)	50

^1^ Oo refers to “olive oil” and Ss to “sesame seeds”.

**Table 3:** Nutritional characterization (humidity, ash, protein, fat, and carbohydrates) of all hummuses in g/100 g on a wet basis. Different letters in the same column represent significant differences.

Hummus	Humidity (g/100 g)	Ash (g/100 g)	Protein (g/100 g)	Fat (g/100 g)	Carbohydrates (g/100 g)
Control	62.94 ± 0.79 ^a^	2.19 ± 0.26 ^a^	7.23 ± 0.04 ^c^	10.05 ± 0.98 ^a^	17.59 ± 0.12 ^c^
*Gelidium corneum*	61.31 ± 0.09 ^a^	2.27 ± 0.13 ^a^	7.13 ± 0.10 ^cd^	8.18 ± 1.26 ^b^	21.11 ± 0.51 ^b^
*Fucus vesiculosus*	55.81 ± 1.88 ^b^	2.30 ± 0.36 ^a^	6.94 ± 0.02 ^d^	9.03 ± 1.15 ^b^	25.92 ± 1.47 ^a^
*Chlorella vulgaris* (heterotrophic)	51.86 ± 0.77 ^c^	2.68 ± 0.05 ^a^	9.70 ± 0.14 ^a^	13.97 ± 0.72 ^a^	21.78 ± 0.15 ^b^
*Chlorella vulgaris* (autotrophic)	51.77 ± 0.36 ^c^	2.39 ± 0.05 ^a^	8.65 ± 0.02 ^b^	13.77 ± 0.13 ^a^	23.42 ± 0.29 ^b^

**Table 4:** Mineral content of all hummuses ^1^. The values in bold represent “SOURCE OF” and the values in bold underlined represent “RICH IN”.

Minerals	Recommended Values in mg/100 g Source of/Rich in	Control	*Gelidium corneum*	*Fucus* *vesiculosus*	*Chlorella* *Vulgaris* Autotrophic	*Chlorella* *Vulgaris* Heterotrophic
Na	-	555.32 ± 3.29 ^b^	521.13 ± 3.36 ^c^	631.89 ± 4.70 ^a^	532.55 ± 13.21 ^bc^	524.34 ± 13.92 ^c^
K	300/600	211.32 ± 1.30 ^d^	**317.75** ± 0.87 ^b^	**361.45** ± 7.31 ^a^	249.04 ± 7.94 ^c^	**319.69** ± 7.16 ^b^
Ca	120/240	**132.50** ± 5.44 ^c^	112.89 ± 2.36 ^d^	**162.15** ± 0.84 ^b^	**197.02** ± 6.58 ^a^	113.58 ± 3.95 ^d^
Mg	57/114	55.98 ± 0.35 ^d^	**72.70** ± 0.55 ^b^	**84.93** ± 1.26 ^a^	**66.87** ± 1.83 ^c^	**73.15** ± 2.00 ^b^
P	105/210	**124.01** ± 2.12 ^b^	**105.58** ± 1.67 ^c^	**106.44** ± 0.45 ^c^	**217.14** ± 6.14 ^a^	**106.24** ± 3.63 ^c^
S	-	96.80 ± 1.11 ^d^	185.86 ± 4.20 ^b^	216.35 ± 8.98 ^a^	126.05 ± 2.68 ^c^	187.04 ± 8.00 ^b^
Fe	2.1/4.2	**2.49** ± 0.04 ^c^	**2.62** ± 0.09 ^c^	**6.49** ± 0.23 ^b^	**8.53** ± 0.25 ^a^	**2.64** ± 0.14 ^c^
Cu	0.15/0.3	**0.36** ± 0.01 ^b^	**0.28** ± 0.01 ^c^	**0.33** ± 0.01 ^b^	**0.43** ± 0.02 ^a^	**0.29** ± 0.02 ^c^
Zn	1.5/3	1.10 ± 0.02 ^bc^	1.05 ± 0.02 ^c^	1.19 ± 0.01 ^b^	**2.66** ± 0.08 ^a^	1.06 ± 0.04 ^c^
Mn	0.3/0.6	**0.63** ± 0.00 ^d^	**0.71** ± 0.01 ^c^	**2.54** ± 0.01 ^a^	**1.19** ± 0.04 ^b^	**0.72** ± 0.02 ^c^

^1^ Different letters in the same row represent significant differences.

**Table 5:** Color properties of hummus (CIELab system). Different letters in the same column represent significant differences.

Samples	L*	a*	b*	ΔE
Control	63.41 ± 0.33 ^a^	3.69 ± 0.13 ^a^	25.50 ± 0.35 ^b^	-
*Gelidium corneum*	56.31 ± 0.57 ^b^	3.17 ± 0.83 ^a^	14.80 ± 0.18 ^e^	12.85
*Fucus vesiculosus*	62.41 ± 0.63 ^a^	0.58 ± 0.11 ^b^	21.62 ± 0.38 ^c^	5.07
*Chlorella vulgaris* (heterotrophic)	34.55 ± 0.87 ^d^	−3.44 ± 0.20 ^c^	16.31 ± 0.36 ^d^	31.11
*Chlorella vulgaris* (autotrophic)	47.94 ± 0.91 ^c^	−4.51 ± 0.08 ^c^	29.45 ± 0.55 ^a^	17.95

**Table 6:** Texture Profile Analysis of Firmness (N) and Adhesiveness (−N.s) for control hummus and hummus with 6% (*w*/*w*) incorporation of seaweeds and microalgae. Different letters in the same column represent significant differences.

Samples	Firmness (N)	Adhesiveness (−N.s)	Cohesiveness
Control	0.32 ± 0.02 ^c^	1.57 ± 0.17 ^c^	0.80 ± 0.02 ^a^
*Gelidium corneum*	0.22 ± 0.01 ^c^	0.80 ± 0.04 ^d^	0.60 ± 0.03 ^c^
*Fucus vesiculosus*	0.28 ± 0.03 ^c^	1.39 ± 0.18 ^c^	0.81 ± 0.03 ^a^
*Chlorella vulgaris* (heterotrophic)	0.82 ± 0.07 ^a^	3.91 ± 0.22 ^a^	0.73 ± 0.02 ^b^
*Chlorella vulgaris* (autotrophic)	0.46 ± 0.01 ^b^	2.46 ± 0.12 ^b^	0.78 ± 0.03 ^ab^

## Data Availability

The original contributions presented in the study are included in the article, further inquiries can be directed to the corresponding author.

## References

[1] Reister E.J., Belote L.N., Leidy H.J. (2020). The Benefits of Including Hummus and Hummus Ingredients into the American Diet to Promote Diet Quality and Health: A Comprehensive Review. Nutrients.

[2] International Center for Advanced Mediterranean Agronomic Studies (CIHEAM) (2012). The Mediterranean Diet for Sustainable Regional Development. MediTERRA.

[3] Andersen N.R., van Deurs Petersen R., Frøst M.B. (2022). Consumer interest in hummus made from different pulses: Effects of information about origin and variety seeking tendency. Int. J. Gastron. Food Sci..

[4] Malcolmson L., Sissons M. (2017). Grains and Pulses Fuel Consumer Trends. Cereal Foods World.

[5] Wallace T.C., Murray R., Zelman K.M. (2016). The Nutritional Value and Health Benefits of Chickpeas and Hummus. Nutrients.

[6] Tosti V., Bertozzi B., Fontana L.V. (2018). Health Benefits of the Mediterranean Diet: Metabolic and Molecular Mechanisms. J. Gerontol. Ser. A.

[7] Shanthakumar P., Klepacka J., Bains A., Chawla P., Dhull S.B., Najda A. (2022). The Current Situation of Pea Protein and Its Application in the Food Industry. Molecules.

[8] United Nations Department of Economic and Social Affairs, Population Division (2022). World Population Prospects 2022: Summary of Results.

[9] Jehn F.U., Dingal F.J., Mill A., Harrison C., Ilin E., Roleda M.Y., James S.C., Denkenberger D. (2024). Seaweed as a Resilient Food Solution After a Nuclear War. Earth’s Future.

[10] Zhao W., Subbiah V., Xie C., Yang Z., Shi L., Barrow C. (2023). Bioaccessibility and Bioavailability of Phenolic Compounds in Seaweed. Food Rev. Int..

[11] Mouritsen O.G., Cornish M.L., Critchley A.T., Pérez-Lloréns J.L. (2023). Chapter 1—History of seaweeds as a food. Applications of Seaweeds in Food and Nutrition.

[12] Ebrahimzadeh S., Biswas D., Roy S., McClements D.J. (2023). Incorporation of essential oils in edible seaweed-based films: A comprehensive review. Trends Food Sci. Technol..

[13] Mouritsen O.G., Rhatigan P., Pérez-Lloréns J.L. (2018). World cuisine of seaweeds: Science meets gastronomy. Int. J. Gastron. Food Sci..

[14] Batista A.P., Gouveia L., Bandarra N.M., Franco J.M., Raymundo A. (2013). Comparison of microalgal biomass profiles as novel functional ingredient for food products. Algal Res..

[15] Sun H., Wang Y., He Y., Liu B., Mou H., Chen F., Yang S. (2023). Microalgae-Derived Pigments for the Food Industry. Mar. Drugs.

[16] Naik B., Mishra R., Kumar V., Mishra S., Gupta U., Rustagi S., Gupta A.K., Preet M.S., Bhatt S.C., Rizwanuddin S. (2024). Micro-algae: Revolutionizing food production for a healthy and sustainable future. J. Agric. Food Res..

[17] Nunes M.C., Ferreira J., Raymundo A. (2023). Volatile fingerprint impact on the sensory properties of microalgae and development of mitigation strategies. Curr. Opin. Food Sci..

[18] United Nations Department of Economic and Social Affairs, Sustainable Division, THE 17 GOALS. https://sdgs.un.org/goals.

[19] Schirmacher H., Elshiewy O., Boztug Y. (2023). That’s not natural! Consumer response to disconfirmed expectations about ‘natural’ food. Appetite.

[20] SUMOL + COMPAL, 75 Anos de História. https://sumolcompal.pt/.

[21] Iberagar, Especialista em Macroalgas Para o Setor dos Hidrocolóides. https://iberagar.com/?lang=pt-pt.

[22] ALGA+, I&D em Consórcio. https://www.algaplus.pt/investigacao/.

[23] Allmicroalgae, Cultivando Soluções Sustentáveis de Microalgas—Allmicralgae. https://www.allmicroalgae.com/pt-pt/.

[24] Cebrián-Lloret V., Martínez-Abad A., López-Rubio A., Martínez-Sanz M. (2023). Sustainable Bio-Based Materials from Minimally Processed Red Seaweeds: Effect of Composition and Cell Wall Structure. J. Polym. Environ..

[25] Morillas-Ruiz J.M., Delgado-Alarcón J.M. (2012). Nutritional analysis of vegetable food with different origins: Evaluation of antioxidant capacity and phenolic total compounds. Nutr. Clínica Dietética Hosp..

[26] Aybar M., Simões S., Sales J.R., Santos J., Figueira D., Raymundo A. (2023). Tenebrio molitor as a Clean Label Ingredient to Produce Nutritionally Enriched Food Emulsions. Insects.

[27] (1999). AACC International, Ash—Basic Method. https://www.cerealsgrains.org/resources/Methods/Pages/08TotalAsh.aspx.

[28] Oliveira S., Torres Pérez M.D., Sousa I., Raymundo A. (2023). 3D-printed Chlorella vulgaris snacks: A contribution to a healthy diet. Front. Food Sci. Technol..

[29] Mota J., Lima A., Ferreira R.B., Raymundo A. (2021). Technological Potential of a Lupin Protein Concentrate as a Nutraceutical Delivery System in Baked Cookies. Foods.

[30] Beltrão M.R., Gouvinhas I., Nunes M.C., Peres J.A., Raymundo A., Barros A.I.R.N.A. (2020). Acorn Flour as a Source of Bioactive Compounds in Gluten-Free Bread. Molecules.

[31] BMG LABTECH, CLARIOstar Plus Microplate Reader—Most flexible. https://www.bmglabtech.com/en/clariostar-plus/.

[32] Zhang Y., Li Y., Ren X., Zhang X., Wu Z., Liu L. (2023). The positive correlation of antioxidant activity and prebiotic effect about oat phenolic compounds. Food Chem..

[33] Reis F.S., Martins A., Barros L., Ferreira I.C.F.R. (2012). Antioxidant properties and phenolic profile of the most widely appreciated cultivated mushrooms: A comparative study between in vxivo and in vitro samples. Food Chem. Toxicol..

[34] Lee H.-G., Nagahawatta D.P., Amarasiri R.P.G.S.K., Jeon Y.-J., Kang M.-C. (2023). Physico-chemical and DPPH-hydroxyl radical scavenging characteristics of crude polysaccharides from *Sargassum thunbergia*. Algal Res..

[35] Gulcin İ., Alwasel S.H. (2023). DPPH Radical Scavenging Assay. Processes.

[36] Zhang M.-Y., Chen S.-L., Lin C.-Y., Zhang H.-X., Zhang T., Zou Z.-M. (2024). New caffeoyl derivatives with potent DPPH radical scavenging activity from Elephantopus tomentosus. J. Asian Nat. Prod. Res..

[37] Olufemi A., Rabiat A.S., Raphael D.O., Mustapha B.O., Salau S. (2024). Phytochemical Profiling and Molecular Docking Investigation of Avocado (Persea Americana Mill. Cultivar Hass) Leaves and Seeds: Implications for Antioxidant Activity and Health Benefits. Res. Sq..

[38] Bulut O., Sönmez Ç., Öktem H.A. (2023). Hindakia tetrachotoma ME03 (Chlorophyta) has high phenolic content, antioxidant capacity, and attenuates H_2_O_2_-induced oxidative stress and apoptosis in human cells. Phycologia.

[39] Neutec Group, LabMaster Neo-Water Activity Meter. https://www.neutecgroup.com/water-activity-measurement/water-activity-labmaster-neo-detail.

[40] Vieira M.R., Simões S., Carrera-Sánchez C., Raymundo A. (2023). Development of a Clean Label Mayonnaise Using Fruit Flour. Foods.

[41] Álvarez-Castillo E., Oliveira S., Bengoechea C., Sousa I., Raymundo A., Guerrero A. (2021). A rheological approach to 3D printing of plasma protein based doughs. J. Food Eng..

[42] Mezger T.G. (2014). Applied Rheology: With Joe Flow on Rheology Road.

[43] Simões S., Carrera Sanchez C., Santos A.J., Figueira D., Prista C., Raymundo A. (2023). Impact of Grass Pea Sweet Miso Incorporation in Vegan Emulsions: Rheological, Nutritional and Bioactive Properties. Foods.

[44] Silva F.G., Passerini A.B.S., Ozorio L., Picone C.S.F., Perrechil F.A. (2024). Interactions between pea protein and gellan gum for the development of plant-based structures. Int. J. Biol. Macromol..

[45] Graça C., Raymundo A., de Sousa I. (2021). Yoghurt and curd cheese addition to wheat bread dough: Impact on in vitro starch digestibility and estimated glycemic index. Food Chem..

[46] Wang Z., Wang L., Yu X., Wang X., Zheng Y., Hu X., Zhang P., Sun Q., Wang Q., Li N. (2024). Effect of polysaccharide addition on food physical properties: A review. Food Chem..

[47] Regulamento 1924/2006 do Parlamento Europeu e do Conselho (20/12/2006) Relativo às Alegações Nutricionais e de Saúde Sobre os Alimentos. https://eur-lex.europa.eu/legal-content/PT/TXT/PDF/?uri=CELEX:32006R1924.

[48] Safi C., Zebib B., Merah O., Pontalier P.-Y., Vaca-Garcia C. (2014). Morphology, composition, production, processing and applications of Chlorella vulgaris: A review. Renew. Sustain. Energy Rev..

[49] Nova P., Pimenta-Martins A., Maricato É., Nunes C., Abreu H., Coimbra M.A., Freitas A.C., Gomes A.M. (2023). Chemical Composition and Antioxidant Potential of Five Algae Cultivated in Fully Controlled Closed Systems. Molecules.

[50] Fang X., Ardehali H., Min J., Wang F. (2023). The molecular and metabolic landscape of iron and ferroptosis in cardiovascular disease. Nat. Rev. Cardiol..

[51] Baj J., Flieger W., Barbachowska A., Kowalska B., Flieger M., Forma A., Teresiński G., Portincasa P., Buszewicz G., Radzikowska-Büchner E. (2023). Consequences of Disturbing Manganese Homeostasis. Int. J. Mol. Sci..

[52] Yilmaz-Ersan L., Suna G. (2024). Comparison of the targeted metabolomics and nutritional quality indices of the probiotic cheese enriched with microalgae. Talanta.

[53] Loughrill E., Wray D., Christides T., Zand N. (2017). Calcium to phosphorus ratio, essential elements and vitamin D content of infant foods in the UK: Possible implications for bone health. Matern. Child Nutr..

[54] Guasti L., Cianferotti L., Pampaloni B., Tonelli T., Mertelli F., Iantomasi T., Brandi M.L. (2023). Evaluation of food and nutrient intake in a population of subjects affected by periodontal disease with different levels of bone mineral density. Front. Endocrinol..

[55] Tang P., Giusti M.M. (2020). Metal Chelates of Petunidin Derivatives Exhibit Enhanced Color and Stability. Foods.

[56] Belattmania Z., Bhaby S., Nadri A., Khaya K., Bentiss F., Jama C., Reani A., Vasconcelos V., Sabour B. (2021). Gracilaria gracilis (Gracilariales, Rhodophyta) from Dakhla (Southern Moroccan Atlantic Coast) as Source of Agar: Content, Chemical Characteristics, and Gelling Properties. Mar. Drugs.

[57] Shewan H.M., Stokes J.R., Smyth H.E. (2020). Influence of particle modulus (softness) and matrix rheology on the sensory experience of ‘*grittiness*’ and ‘*smoothness*’. Food Hydrocoll..

[58] Zhang Z.-H., Wang L.-H., Zeng X.-A., Han Z., Brennan C.S. (2019). Non-thermal technologies and its current and future application in the food industry: A review. Int. J. Food Sci. Technol..

[59] Batista A.P., Nunes M.C., Raymundo A., Gouveia L., Sousa I., Cordobés F., Guerrero A., Franco J.M. (2011). Microalgae biomass interaction in biopolymer gelled systems. Food Hydrocoll..

[60] Kaur M., Singh N. (2005). Studies on functional, thermal and pasting properties of flours from different chickpea (*Cicer arietinum* L.) cultivars. Food Chem..

[61] Bertsch P., Böcker L., Mathys A., Fischer P. (2021). Proteins from microalgae for the stabilization of fluid interfaces, emulsions, and foams. Trends Food Sci. Technol..

[62] Paul A.A., Kumar S., Kumar V., Sharma R. (2020). Milk Analog: Plant based alternatives to conventional milk, production, potential and health concerns. Crit. Rev. Food Sci. Nutr..

[63] Grossmann L., Hinrichs J., Goff H.D., Weiss J. (2019). Heat-induced gel formation of a protein-rich extract from the microalga *Chlorella sorokiniana*. Innov. Food Sci. Emerg. Technol..

[64] Bernaerts T.M.M., Gheysen L., Foubert I., Hendrickx M.E., Van Loey A.M. (2019). The potential of microalgae and their biopolymers as structuring ingredients in food: A review. Biotechnol. Adv..

[65] Braga A.R.C., Nunes M.C., Raymundo A. (2023). The Experimental Development of Emulsions Enriched and Stabilized by Recovering Matter from Spirulina Biomass: Valorization of Residue into a Sustainable Protein Source. Molecules.

[66] Wang M., Yin Z., Sun W., Zhong Q., Zhang Y., Zeng M. (2023). Microalgae play a structuring role in food: Effect of *spirulina platensis* on the rheological, gelling characteristics, and mechanical properties of soy protein isolate hydrogel. Food Hydrocoll..

[67] Cebrián-Lloret V., Martínez-Abad A., López-Rubio A., Martínez-Sanz M. (2024). Exploring alternative red seaweed species for the production of agar-based hydrogels for food applications. Food Hydrocoll..

[68] Oprea O.B., Tolstorebrov I., Claussen I.C., Sannan S., Apostol L., Moșoiu C., Gaceu L. (2023). Potential for Saccharina latissima Flour as a Functional Ingredient in the Baking Sector. Foods.

[69] Bose I., Nousheen, Roy S., Yaduvanshi P., Sharma S., Chandel V., Biswas D. (2023). Unveiling the Potential of Marine Biopolymers: Sources, Classification, and Diverse Food Applications. Materials.

